# Supplementary material for: Climate effects on archaic human habitats and species successions
Source: Nature. 2022 Apr 13;604(7906):495–501. doi: 10.1038/s41586-022-04600-9 (PMC9021022; doi:10.1038/s41586-022-04600-9)
Supplement: Supplementary file 1 — Reporting Summary [file 41586_2022_4600_MOESM1_ESM.pdf]

nature portfolio

Corresponding author(s): Axel Timmermann

Last updated by author(s): Feb 20, 2022

Reporting Summary

Nature Portfolio wishes to improve the reproducibility of the work that we publish. This form provides structure for consistency and transparency in reporting. For further information on Nature Portfolio policies, see our Editorial Policies and the Editorial Policy Checklist.

Statistics

For all statistical analyses, confirm that the following items are present in the figure legend, table legend, main text, or Methods section.

n/a	Confirmed	
☒	☐	The exact sample size (*n*) for each experimental group/condition, given as a discrete number and unit of measurement
☒	☐	A statement on whether measurements were taken from distinct samples or whether the same sample was measured repeatedly
☐	☒	The statistical test(s) used AND whether they are one- or two-sided<br>*Only common tests should be described solely by name; describe more complex techniques in the Methods section.*
☒	☐	A description of all covariates tested
☒	☐	A description of any assumptions or corrections, such as tests of normality and adjustment for multiple comparisons
☒	☐	A full description of the statistical parameters including central tendency (e.g. means) or other basic estimates (e.g. regression coefficient) AND variation (e.g. standard deviation) or associated estimates of uncertainty (e.g. confidence intervals)
☐	☒	For null hypothesis testing, the test statistic (e.g. *F*, *t*, *r*) with confidence intervals, effect sizes, degrees of freedom and *P* value noted<br>*Give P values as exact values whenever suitable.*
☒	☐	For Bayesian analysis, information on the choice of priors and Markov chain Monte Carlo settings
☒	☐	For hierarchical and complex designs, identification of the appropriate level for tests and full reporting of outcomes
☒	☐	Estimates of effect sizes (e.g. Cohen's *d*, Pearson's *r*), indicating how they were calculated

Our web collection on statistics for biologists contains articles on many of the points above.

Software and code

Policy information about availability of computer code

Data collection	The climate model simulations were conducted with the CESM1.2 model on the IBS/ICCP XC50-LC supercomputer Aleph, The computer code of the CESM1.2 model can be downloaded from https://www.cesm.ucar.edu/models/cesm1.2/.
Data analysis	The data analysis of the CESM1.2 simulations was conducted with the Climate Diagnostic Operators https://code.mpimet.mpg.de/projects/cdo/. The habitat suitability model was derived using our own computer codes which were developed for the software Matlab R2014b. Our codes will be shared with interested users on https://climatedata.ibs.re.kr. Maps in Fig. 1, Extended Data Figs. 4, 9, 10, 11 were generated in the matlab package m_map, Pawlowicz, R., 2020. "M_Map: A mapping package for MATLAB", version 1.4m, http://www.eoas.ubc.ca/~rich/map.html. The map in Fig. 4 was generated using the freely-available software Paraview https://www.paraview.org

For manuscripts utilizing custom algorithms or software that are central to the research but not yet described in published literature, software must be made available to editors and reviewers. We strongly encourage code deposition in a community repository (e.g. GitHub). See the Nature Portfolio guidelines for submitting code & software for further information.

Data

Policy information about availability of data

All manuscripts must include a data availability statement. This statement should provide the following information, where applicable:

- Accession codes, unique identifiers, or web links for publicly available datasets
- A description of any restrictions on data availability
- For clinical datasets or third party data, please ensure that the statement adheres to our policy

The CESM1.2 data and the calculated hominin habitat suitability data will be shared on the climate data server https://climatedata.ibs.re.kr. The database of hominin remains, and artefacts generated and used here is provided as Supplementary Table 1. There are no restriction on data usage and access.

Field-specific reporting

Please select the one below that is the best fit for your research. If you are not sure, read the appropriate sections before making your selection.

☐ Life sciences ☐ Behavioural & social sciences ☒ Ecological, evolutionary & environmental sciences

For a reference copy of the document with all sections, see nature.com/documents/nr-reporting-summary-flat.pdf

Ecological, evolutionary & environmental sciences study design

All studies must disclose on these points even when the disclosure is negative.

Study description	Using the Mahalanobis distance approach, a new climate computer model simulation covering the past 2 million years is combined with an extensive hominin fossil and archaeological data compilation (based on previously published sources) to derive a new climate envelope model. The climate envelope model is forced with the climate model input to estimate the time evolution of habitat suitability for 6 hominin species on a spatial mesh of 1 degree x 1 degree and covering the last 2 million years.
Research sample	The fossil and archaeological data compilation used in this study is based on Raia, P., Mondanaro, A., Melchionna, M., Di Febbraro, M. & Diniz-Filho, J. A. F. Past Extinctions of Homo Species coincided with increased vulnerability to Climatic Change. One Earth 3, 1-11 (2020) and extended and updated with new published data sources. Our dataset includes 3246 entries for 6 hominin species during the past 2 million years, each characterized by their latitude, longitude, age and age uncertainties. All data points have been compiled from previously published studies. The climate model used in this study is the Community Earth System Model, version 1.2 in in 3.75×3.75 degree horizontal resolution. Data from this climate model are further downscaled to a 1x1 degree horizontal grid to account for orographic effects. The 6 hominin species (Homo sapiens, Homo neanderthalensis, Homo heidelbergensis, Homo erectus, Homo ergaster, Homo habilis) were chosen to capture key aspects in the evolution of the genus homo between 2 million years ago to 30,000 years ago. The CESM1.2 climate model was chosen for its numerical efficiency (300 simulation years per day on Cray XC50) and its well-documented realistic presentation of the climate system under present-day conditions. Our study focuses on the past 2 million years, due to the availability of the computer model simulation data.
Sampling strategy	The fossil and archaeological data were selected from the vast anthropological and archaeological literature following simple criteria: data samples must be attributable to 1 or 2 (out of the 6) species, age estimates and respective uncertainties must be provided in the original reference, age of samples must cover the time period from 2 million years ago to 30,000 years ago. Based on these criteria our extensive literature search yielded 3246 samples.
Data collection	The novel CESM1.2 computer model simulation for the last 2 million years was conducted by Dr. Kyungsook Yun (co-author).
Timing and spatial scale	The climate model simulation covers the period from 2 million years ago to early Holocene. We therefore concentrate our data analysis on the period 2 Ma - 0.03 Ma. The climate model simulation does not have any gaps. The age gaps for the fossil/ archaeological samples are provided in Supplementary Data 1. Data samples were selected only for Africa and Eurasia, because Australia and the Americas are not a subject of our paper. Temporal resolution: The model simulation output was sub-sampled temporally for the analysis using monthly stratified 1000-year means. This approach is justified, because our study focuses on orbital-scale changes in climate with a minimum timescale of 21,000 years (precessional cycle). Spatial scale: 3.75×3.75 degree horizontal resolution output from the climate model is further downscaled to a 1x1 degree horizontal grid to account for orographic effects and allow for a better comparison with the hominin dataset.
Data exclusions	The entire climate model simulation covering the orbital history of the past 2 million years is included. Our hominin database only includes fossil and archaeological sites that have good chronological constraints. Undated specimen are excluded in our analysis.
Reproducibility	Our study is not based on experimental data, but on numerical simulations, which are highly reproducible within the computational accuracy on other computing platforms.
Randomization	To capture the effect of fossil and archaeological age uncertainties, the climate envelope model links fossil datapoints to climate model data with 100 different ages obtained from a random distribution that spans the most likely age of the hominin data and their corresponding age uncertainties. The climate niche model therefore accounts for the age uncertainties of the hominin record. To further test the effect of climate mean state, variability and exact trajectory on the climate envelope model, we have applied 2 randomization methods: a) 4 dimensional climate data vector was scrambled randomly in time while keeping the 4-dimensional climate covariance intact; b) 4-dimensional climate data vector components are scrambled randomly in time and independently from each other, which destroys the climate co-variance structure, but maintains the average climate state.
Blinding	Blinding is not applicable to our numerical model simulations, because the same computer code will generate the same simulation on other computing platforms, which use the same numerical precision.

Did the study involve field work? ☐ Yes ☒ No

Reporting for specific materials, systems and methods

We require information from authors about some types of materials, experimental systems and methods used in many studies. Here, indicate whether each material, system or method listed is relevant to your study. If you are not sure if a list item applies to your research, read the appropriate section before selecting a response.

Materials & experimental systems

n/a	Involved in the study
☒	☐ Antibodies
☒	☐ Eukaryotic cell lines
☒	☐ Palaeontology and archaeology
☒	☐ Animals and other organisms
☒	☐ Human research participants
☒	☐ Clinical data
☒	☐ Dual use research of concern

Methods

n/a	Involved in the study
☒	☐ ChIP-seq
☒	☐ Flow cytometry
☒	☐ MRI-based neuroimaging
